# Retroperitoneal Mass Masquerading as Failure to Thrive in a 91-year-old Woman

**DOI:** 10.7759/cureus.1831

**Published:** 2017-11-08

**Authors:** Gabriel O Ologun, Noel Yarze, David Bertsch, Joseph Mwesige

**Affiliations:** 1 General Surgery, Guthrie Clinic/Robert Packer Hospital; 2 Family Medicine, Guthrie Clinic/Robert Packer Hospital; 3 Surgical Oncology, Guthrie Clinic/Robert Packer Hospital; 4 Medicine, Guthrie Clinic/Robert Packer Hospital

**Keywords:** abdominal mass, failure to thrive, palliative care, retroperitoneal mass

## Abstract

Failure to thrive (FTT) is a state of overall decline. Patients often present with weight loss, poor appetite, malnutrition, and decreased physical functioning. The etiology is multifactorial including chronic diseases, functional impairments, and acute illnesses. Evaluation for reversible causes is paramount, and treatment is aimed at maintaining or improving functional status. We present a case of a 91-year-old woman with a retroperitoneal mass that was found on workup for failure to thrive.

## Introduction

Failure to thrive (FTT) as a syndrome of weight loss, decreased appetite and poor nutrition, and inactivity; often accompanied by dehydration, depressive symptoms, impaired immune function, and low cholesterol [1]. Physicians should recognize the diagnosis of failure to thrive as a key step in the decision making in the care of an elderly person. We present a case of a 91-year-old woman with a retroperitoneal mass masquerading as failure to thrive. Informed consent was obtained for the case report, images, and for publication.

## Case presentation

**Figure 1:**
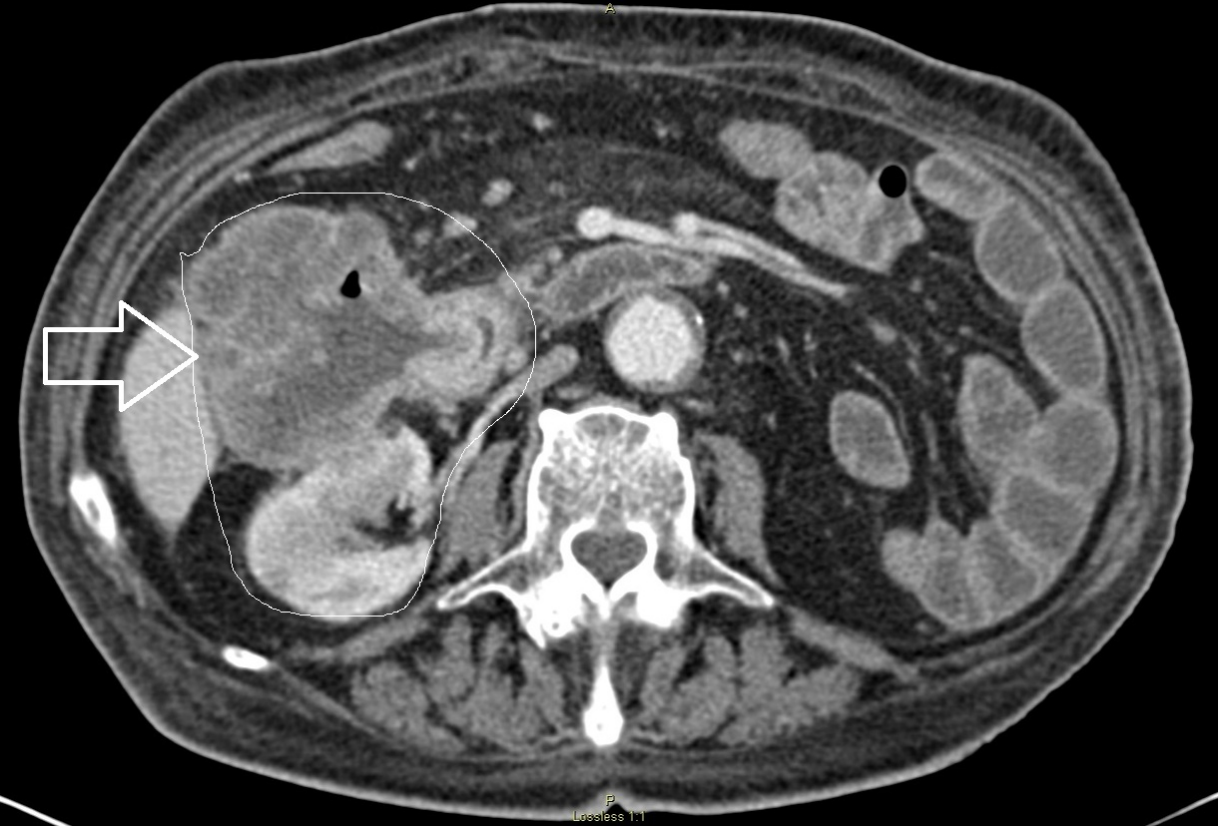
Computed tomography (CT) scan of abdomen/pelvis, axial view showing a right colon (hepatic flexure) mass invading the second portion of the duodenum and the right kidney. Areas of interest outlined, and arrow pointing at the hepatic flexure mass.

A 91-year-old female with a medical history of bilateral breast cancer, status post mastectomy and chemotherapy, iron deficiency anemia, hypertension, appendectomy, cholecystectomy, and dementia who lived in a long-term care facility. She had decreased appetite for several months, weight loss, intermittent nausea and vomiting, and was sedentary. Laboratory studies obtained at that time were significant for anemia, but with normal thyroid function studies. She had a 40 pack-year history of smoking, quit smoking over 27 years ago. Her home medications included: donepezil, metoprolol, hydralazine, Tylenol, colace, and cyanocobalamin. The patient was admitted to our hospital with the diagnosis of FTT. Laboratory investigation revealed white blood cell (WBC) 16 K/uL, hemoglobin 8.4 g/dL, platelets 195 K/uL, international normalization ratio (INR) 1.24; complete metabolic panel shows serum sodium 135 mmol/L, potassium 3.7 mmol/L, chloride 103 mmol/L, bicarbonate 24 mmol/L, glucose 94 mg/dL, blood urea nitrogen 10 mg/dL, creatinine 0.6 mg/dL, calcium 7 mg/dL, total protein 4.7 g/dL, albumin 2 g/dL, alkaline phosphatase (ALP) 57 U/L, aspartate aminotransferase (AST) 10 U/L, alanine aminotransferase (ALT) 18 U/L, total bilirubin 0.2mg/dL. Evaluation with computed tomography (CT) scan of the chest and abdomen/pelvis revealed a right hepatic flexure mass invading the second portion of the duodenum and the right kidney (Figure 1), with no clinical evidence of bowel obstruction. Carcinoembryonic antigen (CEA) 1090 ng/ml (reference- less than 3 ng/mL).

Surgical oncology team was consulted to evaluate the patient. On examination the patient had a nasogastric tube in place with bilious output, palpable non-tender mass in her right upper abdomen; the remainder the examination was unremarkable. The CT findings and management options were discussed with the patient, including a palliative care option. Patient opted for surgical intervention. After appropriate preoperative management, the patient was taken to the operating room for an exploratory laparotomy for en bloc resection of the mass for presumed colon cancer with invasion of duodenum and kidney. Intraoperatively, ascites was noted, the bowel was edematous, and the mass was fixed to the right retroperitoneum, kidney and duodenum; the overall findings suggested major resection would probably have a poor outcome. Therefore, gastrojejunostomy and ileocolic bypasses were created to prevent progression to clinical obstruction. Postoperatively, the patient had an uneventful recovery. Operative findings were discussed with the patient and she decided to pursue palliative care management. She was discharged to her long-term care facility on postoperative day eight.

## Discussion

The United States National Institute of Aging describes failure to thrive (FTT) as a syndrome of weight loss, decreased appetite, poor nutrition, and inactivity; often accompanied by dehydration, depressive symptoms, impaired immune function, and low cholesterol [1]. However, the four hallmark symptoms of FTT are depression, cognitive impairment, malnutrition, and decreased physical functioning [2]. Acute illnesses, and not social factors, were the primary reason for admission among elderly patients given a diagnosis of FTT [1].

Dementia has a variable life expectancy, progressing to death over 3–12 years and affects over five million Americans and 36 million people worldwide, creating substantial burdens for their caregivers of which FTT is a commonly associated syndrome [3]. It is therefore unlikely, based on the natural history, that progressive dementia was attributable to our patient’s condition.

This raises the question of whether FTT is a resignation to failed psychosocial interventions versus a tip of the iceberg to an underlying acute medical condition. It is, therefore, imperative to not only look for psychosocial causes of FTT but acute medical conditions.

Advance care planning is therefore valuable for conditions such as FTT, and this has been a priority since the 1990 Patient Self Determination Act with the goal of enhancing decision making at end of life [4]. Prognostication being a part of advance care planning evokes disease and psychosocial discussions which impact patient care/interventions as it did in our patient.

Unfortunately, there seems to be a discordance between living will (LW) and end-of-life wishes possibly grounded by the disparate scenarios painted by the LW and the reality of end-of-life. Our patient struggled with this decision, however, the knowledge of the advanced nature of her colon mass and poor prognosis eased the decision-making process.

## Conclusions

Prognostication during advanced care planning is essential when setting up a LW. This will help minimize the mismatch of case scenarios during the establishment of the LW and the reality of circumstances associated with the end of life. Advanced care planning might require routine revisiting due to the complexities attributable to disease conditions and patient indecision with end of life care planning.

## References

[1] Kumeliauskas L, Fruetel K, Holroyd-Leduc JM (2013). Evaluation of older adults hospitalized with a diagnosis of failure to thrive. Can Geriatr J.

[2] Sera L, Holmes HM, McPherson ML (2014). Prescribing practices in hospice patients with adult failure to thrive or debility. Prog Palliat Care.

[3] Hanson LC, Ersek M, Lin FC (2013). Outcomes of feeding problems in advanced dementia in a nursing home population. J Am Geriatr Soc.

[4] Winter L, Parks SM, Diamond JJ (2010). Ask a different question, get a different answer: why living wills are poor guides to care preferences at the end of life. J Palliat Med.

